# Monomeric ß-amyloid interacts with type-1 insulin-like growth factor receptors to provide energy supply to neurons

**DOI:** 10.3389/fncel.2015.00297

**Published:** 2015-08-07

**Authors:** Maria L. Giuffrida, Marianna F. Tomasello, Giuseppe Pandini, Filippo Caraci, Giuseppe Battaglia, Carla Busceti, Paola Di Pietro, Giuseppe Pappalardo, Francesco Attanasio, Santina Chiechio, Silvia Bagnoli, Benedetta Nacmias, Sandro Sorbi, Riccardo Vigneri, Enrico Rizzarelli, Ferdinando Nicoletti, Agata Copani

**Affiliations:** ^1^National Research Council, Institute of Biostructure and BioimagingCatania, Italy; ^2^PhD Program in Neuropharmacology, University of CataniaCatania, Italy; ^3^Department of Clinical and Molecular Biomedicine, University of CataniaCatania, Italy; ^4^Department of Drug Sciences, University of CataniaCatania, Italy; ^5^IRCCS Associazione Oasi Maria S.S., Institute for Research on Mental Retardation and Brain AgingTroina, Italy; ^6^Department of Molecular Pathology, Neuropharmacology Unit, IRCCS NeuromedPozzilli, Italy; ^7^NEUROFARBA, University of FlorenceFlorence, Italy; ^8^Department of Human Physiology and Pharmacology, University “La Sapienza”Rome, Italy

**Keywords:** Alzheimer's disease, ß-amyloid, glucose, IGF-IR, Glut3

## Abstract

ß-amyloid (Aß_1−42_) is produced by proteolytic cleavage of the transmembrane type-1 protein, amyloid precursor protein. Under pathological conditions, Aß_1−42_self-aggregates into oligomers, which cause synaptic dysfunction and neuronal loss, and are considered the culprit of Alzheimer's disease (AD). However, Aß_1−42_ is mainly monomeric at physiological concentrations, and the precise role of monomeric Aß_1−42_ in neuronal function is largely unknown. We report that the monomer of Aß_1−42_ activates type-1 insulin-like growth factor receptors and enhances glucose uptake in neurons and peripheral cells by promoting the translocation of the Glut3 glucose transporter from the cytosol to the plasma membrane. In neurons, activity-dependent glucose uptake was blunted after blocking endogenous Aß production, and re-established in the presence of cerebrospinal fluid Aß. APP-null neurons failed to enhance depolarization-stimulated glucose uptake unless exogenous monomeric Aß_1−42_ was added. These data suggest that Aß_1−42_ monomers were critical for maintaining neuronal glucose homeostasis. Accordingly, exogenous Aß_1−42_ monomers were able to rescue the low levels of glucose consumption observed in brain slices from AD mutant mice.

## Introduction

Sporadic age-related form of AD is the most frequent yet incurable type of dementia. Difficulties in advancing disease understanding and clinical interventions partly depend on complex interactions between disease determinants, namely the toxic ß-amyloid_1−42_ (Aß_1−42_) species (Selkoe, 2011), and age-related risk factors, including the decline of insulin-like growth factor 1 (IGF-1) functions (Piriz et al., 2011), and the occurrence of peripheral insulin resistance or diabetes (Jayaraman and Pike, 2014). These intervening factors, among others, might progressively overcome the ability of the brain cognitive reserve to buffer the impact of the pathology on cognitive functions. Specifically, Stranahm and Mattson have hypothesized that the cognitive reserve relies on insulin/neurotrophic factor signaling and glucose metabolism that all set the brain metabolic efficiency (Stranahan and Mattson, 2012). The AD brain shows impairments of insulin/IGF-1 signaling (de la Monte, 2012) and glucose metabolism (Caselli et al., 2008), which only in some cases depend on a comorbid systemic disease (de la Monte, 2012), and are largely unexplained. Talbot et al. (2012) have provided direct evidence that AD brain tissue is insulin resistant in the absence of diabetes, and that histological biomarkers of insulin resistance co-localize with Aß plaques. Interestingly, in the same study, they found a strong resistance of type-1 IGF receptor (IGF-IR) to ligand activation even in brain regions where amyloid plaques are found in a very late stage of AD (Talbot et al., 2012). It remains unknown whether and how this last finding is relevant to the pathophysiology of AD and is related to the low rate of brain glucose metabolism that starts decades before the clinical onset of dementia (Reiman et al., 2004; Mosconi et al., 2006; Caselli et al., 2008). Here we provide evidence that, both in native and recombinant systems, IGF-IRs can be activated by the monomer of Aß_1−42_, the predominant form of the protein at physiological concentrations (Nag et al., 2011). In recombinant cells, monomeric Aß_1−42_ acts as a positive allosteric modulator of IGF-IRs endowed with a small intrinsic efficacy, thereby enhancing IGF-1 effects. Through IGF-IR activation, Aß_1−42_ monomers control glucose uptake in neurons under both basal and depolarizing conditions. Interestingly, Aß_1−42_ monomers rescue the low levels of glucose consumption observed in brain slices from AD transgenic mice, which are resistant to IGF-1 stimulation. We suggest that a defective IGF-IR signaling contributes to AD progression *via* a disease-specific mechanism involving the loss of receptor activation by Aß monomers, which become depleted when pathological aggregates are formed.

## Materials and methods

### Synthesis of pentapeptides

Pentapeptides (KLVFF, VFLKF, klvff, ffvlk) were synthesized by means of microwave-assisted solid phase peptide synthesis on a CEM “Liberty” peptide synthesizer using standard 9-fluorenylmethoxycarbonyl (Fmoc) chemistry. Peptides were cleaved off from the solid support using a mixture of Trifluoro-acetic acid (TFA)/water (H_2_O)/tri-isopropyl-silane (TIS) 95/2.5/2.5 (v/v/v), then precipitated with cold freshly distilled diethyl ether. Crude peptides were purified by preparative RP-HPLC. Samples identity was confirmed by ESI-MS (Calculated mass for C_37_H_55_N_7_O_6:_ 693.42; Observed [M+H]^+^: 694.58).

To control for KLVFF effects, in addition to the retroinverse ffvlk, which maintains the overall spatial topology of KLVFF, we chose to synthesize both the scrambled peptide, VFLKF, and the D-enatiomer klvff. The latter is very likely to bind Aß_1−42_ as the parent KLVFF (Chalifour et al., 2003), thus ruling out the possibility that KLVFF acts by stabilizing endogenous Aß monomers.

### Peptide sample preparation

Aß_1−42_ and Aß_1−16_ were purchased from Bachem Distribution Services GmbH, Germany. Aß_17−42_ was purchased from Innovative Peptide Solutions, Germany. All peptides were dissolved in trifluoroacetic acid (TFA) at a concentration of 1 mg/ml and sonicated for 10 min. TFA was removed by gentle streaming of argon. Peptides were then dissolved in 1,1,1,3,3,3-hexa-fluoro-2-propanol (HFIP) and incubated at 37°C for 1 h. Following argon streaming, peptides were dissolved again in HFIP, lyophilized and then resuspended in 5 mM anhydrous dimethyl sulfoxide (DMSO) prior to dilution to 100 μM in ice-cold cell culture medium DMEM-F12.

### Circular dichroism measurements

CD spectra were recorded at 37°C under a constant nitrogen flow on a JASCO model J-810 spectropolarimeter, equipped with a Peltier thermostatted cell holder. CD spectra were run in the far-UV region (200–260 nm) using 1 cm path length cuvettes. CD spectra were acquired every 30 min over a time course of 1200 min. Buffer contribution to the CD intensity was subtracted from peptide CD spectra.

### Thioflavin T (ThT) fluorescence measurements

Fluorescence measurements were performed on a Perkin Elmer LS 55 spectrophotofluorimeter equipped with a thermostatic cell holder. The experiments were carried out at 37°C using a 1 cm light path quartz. ThT (45 μM) emission fluorescence was followed for 1200 min by monitoring the increase in the dye intensity at 480 nm, with a 440 nm excitation wavelength. The excitation and emission slit widths were set at 5 nm.

### Rayleigh scattering measurements

Rayleigh scattering measurements were performed on a Perkin Elmer LS 55 spectrophotofluorimeter at 37°C in a 1 cm path-length cell. Peptide samples were excited at 400 nm and scattering was monitored for 1200 min at 400 nm. Both excitation and emission slits were fixed at 5 nm.

### IGF-IR and IR phosphorylation assay

Clones of R^−^ cells (3T3-like mouse fibroblasts with a disrupted IGF-IR gene), stably transfected with either the human IGF-IR (R^+^) or the human IR-A cDNA (R^−^ IR-A), were obtained as previously described (Pandini et al., 2002). Cell lysates from R^−^IR-A cells or R^+^ cells (40 μg protein/well) were immunocaptured in Maxisorp Break-Apart immunoplates (Nunc) coated with antibodies MA-20 (Novus Biologicals), which recognizes the IR α-subunit, and αIR-3 (Calbiochem), which recognizes the IGF-IR α-subunit, at a concentration of 2 and 1 μg/ml, respectively, in 50 mm sodium bicarbonate (pH 9.0) overnight at 4°C. After washing, the immunocaptured receptors were incubated with increasing concentrations of either porcin insulin (Sigma-Aldrich) or recombinant human IGF-1 (PeproTech) (in 50 mm HEPES-buffered saline (pH 7.6), 150 mm NaCl, 0.1% TritonX 100, BSA 0.05%, containing 10 μM ATP, 10 mM MgCl_2_ and 2 mM MnCl_2_) in the presence or absence of mAß_1−42._After 2 h at RT, the plates were washed and the captured phosphorylated proteins were incubated with biotin-conjugated anti-phosphotyrosine antibody 4G10 (0.3 μg/ml in 50 mm HEPES (pH 7.6), 150 mm NaCl, 0.05% Tween 20, 1% BSA, 2 mm sodium orthovanadate, 1 mg/ml bacitracin) for 2 h at RT and then with peroxidase-conjugated streptavidin. The peroxidase activity was determined colorimetrically by using the TMB microwell peroxidase substrate system (KPL). The reaction was stopped by the addition of 1.0 m H_3_PO_4_, and the absorbance was measured at 450 nm.

### Neuronal cultures: preparation and treatments

Animal care and experimentation was in accordance with institutional guidelines. Cultures of pure cortical neurons were obtained from rats at embryonic day 15 as described previously (Giuffrida et al., 2009). Cultures of mixed cortical cells, contaning both neurons and glia, were obtained from rats at embryonic day 17 and grown onto poly-D-lysine coated 16 mm multiwell vessels (4 × 10^5^ cells/well) as described previously (Giuffrida et al., 2009). Mature cultures, at 14–16 days *in vitro* (DIV), were used for the study. All experiments were performed always after extensive washing of the cultures to avoid any interference with serum IGF-1/IGFBP3/ALS ternary complexes.

Mature pure neuronal cultures at 7 DIV were deprived from insulin and, where required, peptide monomers were added and maintained for 48 h. AG1024 (100 nM) and PPP (500 nM) were applied for 30 min before insulin deprivation. Neuronal survival was assessed by 3-(4,5-dimethylthiazol-2-yl)-2,5-diphenyl tetrazolium bromide (MTT) reduction assay.

Mixed cortical cultures at maturation were exposed to 300 μM NMDA for 10 min at room temperature in a HEPES-buffered salt solution. Neuronal toxicity was examined 24 h later by light microscopy and quantified after staining with trypan blue (0.4% for 5 min). Stained neurons were counted from three-random fields/well. Peptide monomers were added in combination with NMDA. Where required, AG1024 (100 nM) and PPP (500 nM) were applied 15 min before the excitotoxic pulse.

IGF-1 release in neuronal cultures was assessed by the IGFBP-blocked IGF-1 ELISA (ALPCO Diagnostics). Cultures were exposed to the washing protocol utilized for all the experiments and IGF-1 content was quantitated in the culture buffer within 2 h. Low levels of IGF-1 were generally found under these conditions (about 0.003 nM). However, IGF-1 levels strictly depended on the experimental condition. For example, a brief NMDA pulse lead to a 5 fold increase in the release (up to 0.017 nM). Since the assay detected total IGF-1 (free and IGFBP-bound), measurements could not provide indications about the active quote of the factor.

Cultures of APP-null neurons were obtained as in Giuffrida et al. (2009) except that cortices from newborn mice (within 12 h from birth) were used. Homozygous APP-null male and female mice (B6.129S7-Apptm1Dbo/J) on a C57BL/6J genetic background were purchased from Charles River, and the colony has been established in the animal house of the University of Catania.

### Imaging of 6-NBDG up-take in neurons by laser scanning confocal microscopy (LSM)

Neurons were plated on glass bottom culture dishes and were used at 6–8 DIV. For the experiments, cultures were rinsed with glucose-free HCSS (120 mmol/l NaCl, 5.4 mmol/l KCl, 1.8 mmol/l CaCl_2_, 20 mmol/l HEPES pH 7.4) and kept for 45 min under glucose deprivation followed by exposure for 30 min to either mAß_1−42_ (100 nM), or recombinant rat IGF-1 (5 ng/ml, R&D Systems). The non-hydrolyzable glucose analog 6-NBDG was allowed to be internalized into neuronal cells at 37°C and 5% CO_2_ for 10 min_._ 6-NBDG^+^ neurons were imaged by using an Olympus FV1000 LSM. Images were captured at 488 excitation/505-550 emission.

### Assessment of 6-NBDG uptake in neurons by cytofluorimetric analysis

Neurons were grown onto 35 mm dishes and the experiments were performed at 7 DIV. Cultures were rinsed in glucose-free HCSS and maintained for 45 min under glucose deprivation followed by exposure to either mAß_1−42_ (100 nM) or IGF-1 (5 ng/ml) for 30 min, or to KCl (40 mM) for 15 min. PPP (500 nM) was added 15 min before mAß_1−42_. When required, a γ-secretase inhibitor (γ-sec-Inhibitor IX, Calbiochem, 100 nM) was added 2 h before glucose deprivation and maintained troughout the experiment. 6-NBDG (100 μM) was added 10 min before ending the experiment by rinsing the cells twice with ice-cold phosphate buffered saline (PBS). Neurons were scraped into ice-cold PBS and maintained at 4°C for the cytofluorimetric analysis (cytomics FC500, Beckman Coulter). 20,000 Events for experimental condition (each in triplicate) were collected.

### Assessment of glucose uptake in neurons exposed to human CSF

Neurons were grown into 96 well plates and the experiments were performed at 7 DIV. Before the experiment, glucose concentration was measured in all CSF samples by glucose meter, and glucose levels were therefore adjusted to the highest measured value. For the experiment, cultures were rinsed in glucose-free artificial CSF (124 mM NaCl, 2.5 mM KCl, 2 mM MgSO_4_, 1.25 mM KH_2_PO_4_, 26 mM NaHCO3, 2.5 mM CaCl_2_) and maintained for 45 min under glucose deprivation. γ-Sec-inhibitor IX (Calbiochem, 100 nM) was added 2 h before glucose deprivation and maintained troughout the experiment. Following deprivation, cultures were shifted into 50 μl CSF/well and preliminary experiments were carried out to assess neuronal viability. Under this condition, a fast excitotoxic event occurred and thereafter all experiments were carried out in the presence of the NMDA receptor antagonist, MK-801 (1 μM). Cultures were maintained in CSF with MK-801 for 40 min, during which no glucose uptake occurred.

Parallel experiments carried out in artificial CSF showed that glucose uptake in response to KCl, at the usual concentration of 40 mM, was blunted by the presence of MK-801 (glucose consumption (mg/dl): 52.75 ± 1.5 and 47.27 ± 1.6–basal vs. KCl, respectively). However, a relevant glucose uptake was observed by rising KCl concentration to 400 mM (glucose consumption (mg/dl): 53.25 ± 2.8 and 38 ± 2.3–basal vs. KCl, respectively).

Neurons were therefore maintained in CSF + MK-801 for 40 min, then glucose consumption was measured in a 5 μl aliquot by glucose meter before adding 400 mM KCl for 15 min. Measurements of glucose consumption were taken again at the end of the pulse, and glucose uptake was calculated with respect to the initial values.

### L6 cell cultures

L6 rat skeletal muscle cells (EACC) were cultured in 5% CO2 in DMEM (Invitrogen) supplemented with 10% fetal bovine serum (FBS) and 1% penicillin/streptomycin. Once the cells reached 80% confluence, they were split (using 0.25% trypsin-EDTA) into fractions and propagated or seeded to be used in the experiments. To induce differentiation into the myotubes that were used for experiments, cells were made confluent and the concentration of FBS was then reduced to 1% for 24–48 h. Cells were passaged biweekly. Passages 10–30 were used for all the experiments.

### Measurement of 6-NBDG uptake in L6 cells

L6 myotubes, seeded in 22 mm glass bottom dishes, were placed in Krebs'Ringer buffer (136 mM NaCl, 20 mM HEPES, 2 mM NaHCO3, 0.5 mM NaH2PO4, 3.6 mM KCl, 0.5 mM MgCl2, and 1.25 mM CaCl2, pH 7.4) without glucose for 45 min. When required, inhibitors were added for 30 min during the glucose starvation time. The stimulation of glucose uptake was obtained by incubating cells for 15 more min with either IGF-1, mAß_1−42_, or KVLFF. Cells were then loaded with 100 μM 6-NBDG for 15 min. The concentration and incubation time were chosen as the best for giving an adequate signal/noise ratio. After the loading period, culture dishes were washed twice and placed on the stage of a FV1000 LSC microscope. Cultures were excited at 488 nm, and 6-NBDG was imaged at 505–550 nm emission wavelength.

### Indirect Glut3 immunofluorescence analysis

To mimic the experimental conditions under which glucose uptake was observed, cells seeded in round coverslips (pure cortical neurons or L6 myotubes) were washed repeatedly and glucose starved for 30 min. When required, PPP was added during the glucose starvation time. Cells were then washed again and stimulated for 10 min with monomeric Aß_1−42_ (100 nM), KCl (40 mM), or KLVFF monomers (100 nM) in the presence of glucose. Cells were then fixed in 2% formaldehyde and permeabilized using 0.1% Triton X-100. Unspecific binding was blocked by 30 min of incubation in 4% bovine serum albumin (BSA) in 0.1% Triton X-100-PBS. Glut-3 was detected by incubating over-night cells with rabbit anti-Glut-3 antibody (1:100, Abcam). Counterstaining was obtained by over-night incubation with mouse anti-actin (1:200, Sigma-Aldrich). After PBS washing, cells were exposed for 1 h at RT to the respective secondary antibody (anti-rabbit AlexaFluor 546 or anti-mouse AlexaFluor 680). Coverslips were mounted with the ProLong Gold antifade mounting medium (Invitrogen) and examined under a FV1000 LSC microscope using the Fluoview Olympus image software.

Imaging was carried out using a 63 Plan-Apo/1.4-NA oil-immersion objective. Standard 3 confocal channel (3 photomultiplier detectors) acquisitions were made by using the following lasers, mounted on a laser combiner: Multi-line Argon laser (457, 488, 515 nm), total 30 mW HeNe-Green laser (543 nm), 1.5 mW HeNe-Red laser (633 nm), 10 mW. Single or multiple optical sections (0.42 μm *z* axis) through the middle of the cells were acquired for each field. The pinhole was adjusted to keep the same size of *z*-optical sections for all the analysis. Sequential mode imaging was performed to ensure that there was no crosstalk between the channels. Ten random fields for each treatment were imaged, with each treatment repeated twice in three separate experiments. Quantitative analysis was carried out using the FV1000 single particle analysis software (release 2). Glut3 signal spread was calculated as follows: for each microscopic field a z-stack series, made up of 20 slices (0.42 μM thickness each), was acquired. Then, the difference between the average fluorescence intensity/pixel, measured as z-projection in the z-stack series, and the average fluorescence intensity/pixel, measured for a single slice corresponding to the middle of the neuron, was calculated.

### Detection of the Glut3 exofacial domain

Specific detection of the N-terminal extracellular domain of Glut3 was achieved by indirect immunofluorescence of non-permeabilized neurons, using a goat antibody raised against a peptide mapping within this region (1:25, Santa Cruz). Glut3 immunoreactivity was revealed with the donkey anti-goat IgG-Texas Red, Santa Cruz). Quantitative analysis was carried out by flow cytometry. Briefly, at the end of each treatment, neurons were harvested, washed once with PBS and pelleted. The cell pellet was fixed by incubation with 200 μl of 2% formaldehyde for 1 h at RT. Fixed cells were blocked with 4% BSA, and stained by 2 h incubation at RT with the exofacial Glut3 antibody. Stained cells were then washed twice, and finally revealed by 1 h incubation with the secondary Texas Red-conjugated antibody. Immunostained samples were checked by cytofluorimetric analysis with a CyFlow® ML flow cytometer system (Partec). Neurons were excited by an air-cooled argon 488 nm laser and Texas Red signal was read on FL3 detector. The data acquired (20,000 cells per sample) were compensated, gated and analyzed using FlowMax software (Partec). Each experimental condition was repeated in triplicate.

### Western blotting analysis

Western blotting analysis for phospho-eIF-4E binding protein (p4EBP1) or phospho-IGF-IR was performed on total protein extracts (50 μg) from differentiated L6 cells treated with IGF-1 (2 ng/ml), mAß_1−42_ (100 nM), or KLVFF (100 nM) for 15 min. Samples were loaded onto 4-12% bis-Tris Glycine gel (NuPAGE, Invitrogen). After separation, proteins were transferred onto a nitrocellulose membrane (Hybond ECL, Amersham Italia) using a transblot semi-dry transfer cell. Membranes were blotted at 4°C o.n. with the following primary antibodies: rabbit anti-p4EBP1 (1:1000, Cell Signaling), rabbit-anti(Y1161)IGF-IR (1:700, Abcam), and mouse anti-ß-actin (1:1000, Sigma Aldrich). Secondary goat anti-rabbit labeled with IRDye 680 (1:14.000 Li-COR Biosciences) and goat anti-mouse labeled with IRDye 800 (1:12.000 Li-COR Biosciences) were used at RT for 45 min. Hybridization signals were detected with the Odyssey Infrared Imaging System (LI-COR Biosciences).

For the analysis of phospho-IRs, cell lysates were obtained from pure neuronal cultures following a 15 min stimulation. Cell lysates (150 μg) were incubated for 2 h at 4°C on a rotating device with 20 μl of the agarose conjugated anti-IR β (500 μg/0.25 ml agarose, Santa Cruz Biotechnology). Immunoprecipitates were pelleted, washed and resuspended in 25 μl of 1x electrophoresis sample buffer containing dithiothreitol. Following SDS-PAGE and transfer, blots were probed with a monoclonal anti-phosphotyrosine antibody (1:1000, 4G10 from Merck Millipore). To search for specific hybridization signals, membranes were incubated with a horseradish peroxidase-conjugated secondary antibody, followed by the SuperSignal chemiluminescent detection system. After probing with the anti-phosphotyrosine antibody, blots were stripped for 15 min with Strip Ablot (EuroClone), and re-probed with anti-IR β (1:1000, Cell Signaling) to control for loading.

Western blotting analysis of phospho-IGF-IR in cultured neurons was performed on 40 μg of total proteins. Membranes were incubated over night at 4°C with the following primary antibodies: rabbit anti-p(Y1161)IGF-IR (1:700, Abcam), and mouse anti-ß-actin (1:1000, Sigma Aldrich). For the detection of hybridization signals, membranes were incubated with secondary goat anti-rabbit labeled with IRDye 680 (1:14.000 Li-COR Biosciences) and goat anti-mouse labeled with IRDye 800 (1:12.000 Li-COR Biosciences) for 45 min at RT. Signals were detected with the Odyssey Infrared Imaging System (LI-COR Biosciences). Alternatively, immunoprecipitation was carried out on neuronal lysates (150 μg) by using the rabbit anti-IGF-IR β (10 μg/mg protein lysate, Cell Signaling Technology).

### Quantitative determination of Aß_1−42_ in neuronal culture supernatants

To mimic the experimental conditions under which 6-NBDG was observed, neuronal cultures were rinsed in glucose-free HCSS and maintained for 30 min under glucose deprivation. KCl (40 mM) was added 15 min before ending the experiment. When required, γ-sec-Inhibitor IX (100 nM) was added 2 h before glucose deprivation. Aß_1−42_ was quantitated in the collected HCSS by using the Wako human/rat Aß_42_ ELISA kit, high-sensitive. This kit detects human/rat Aß_*x*−42_ in the 0.1–20 pmol/L range, with a sensitivity of 0.024 pmol/L; 100 μl of undiluted cell supernatants from equivalent cultures were used for the assay.

#### Assessment of insulin release in pancreatic INS-1E cells

Rat INS-1E ß-cells were maintained in RPMI-1640 medium with 11.1 mmol/l D-glucose, supplemented with 10% FBS, 100 U/ml penicillin, 100 μg/ml streptomycin, 10 nmol/l HEPES, 2 nmol/l L-glutamine, 1 nmol/l sodium pyruvate and 50 μmol/l β-mercaptoethanol. Cells were grown in T-75 flask at 37°c and 5% CO_2_and passaged every 5 days by using 0.05% trypsin-EDTA. For the experiments, cells were plated onto 24-well plates at a density of 0.5 × 10^6^ cells/well and grown to 100% confluence. 18 h Before the experiment, growing medium was replaced with fresh medium containing 5 mmol/l glucose. Insulin secretion assay was performed in HEPES balanced salt solution (HBSS) (114 mmol/l NaCl, 4.7 mmol/l KCl, 1.2 mmol/l KH_2_PO_4_, 1.16 mmol/l MgSO_4_, 20 mmol/l HEPES, 2.5 mmol/l CaCl_2_, 25.5 mmol/l NaHCO_3_, 0.2% bovine serum albumin), pH 7.2. Cells were washed and maintained in 3 mM glucose HBSS for 2 h before the switch in 15 mM glucose either in the absence or in the presence of mAß_1−42_ (100 nM) and KLVFF (100 nM). When required, PPP (500 nM) was added 15 min before the switch. The supernatant was collected 15 min after switching, and the total insulin content was determined by the use of rat/mouse Insulin Enzyme Linked Immunosorbent Assay (ELISA) kit (Millipore).

### Intra-peritoneal glucose tolerance test (GTT)

CD1 male mice (33-35 g body weight), C57BL/6J male mice (8 weeks of age), and transgenic B6.129S7-Apptm1Dbo/J male mice (APP-null, 8 weeks of age) were housed up to five for cage and fasted for 16 h prior to the test. For the GTT, a solution of glucose (20% in 0.9% NaCl) was administered by intra-peritoneal (i.p.) injection (100 μl/10 g body weight) and blood glucose was measured at different time points during the following 4 h. Blood glucose was obtained from the paw and measured by using the blood glucose meter One Touch Vita, J&J. Assuming a plasma volume of 1.5 ml/mouse, stock solution of Aß_1−42_ monomers, Ac-KLVFF-NH_2_ monomers or recombinant rat IGF-1 were prepared freshly in saline so to reach the plasma concentrations of 100 nM for monomers, and 2 ng/ml for IGF-1. After basal blood glucose measurements, where appropriate, monomers and IGF-1 were i.p. injected 5 min before or 15 min after glucose loading. Animal care and experimentation was in accordance with institutional guidelines.

### Ex-vivo brain stimulation

Heterozygous TgCRND8 male mice on a 129/SVE genetic background were initially obtained from the Center for Research in Neurodegenerative Diseases, University of Toronto, Ontario, Canada. The colony has been established in the Animal House of the University of Catania, Italy. Animal care and experimentation was in accordance with institutional guidelines. Female TgCRND8 and non-Tg littermate mice at 12 months or 8 weeks of age were used for the experiment. Mice were sacrified by CO_2_ asphyxiation, brains were removed and forebrains were sliced with a McIlwain tissue chopper. Slices were washed three times (for a total time of 30 min) with oxygenated Krebs-Ringer (K-R) solution (118 mM NaCl, 25 mM Hepes, 4.8 mM KCl, 1.3 mM CaCl2, 1.2 mM KH2PO4, 1.2 mM MgSO4; 25 mM NaHCO3; 10 mM glucose), and 300 μl of the slice suspension was diluted in single tubes in a total K-R volume of 700 μl. Incubation was carried out at 37°C for 1 h in an aerated (95% O_2_, 5% CO_2_) water bath. Stimulation was performed with either 1 nM IGF-1 or 100 nM Aß_1−42_ monomers and terminated by adding 2 ml of ice-cold K-R. Slices were collected by centrifugation, lysed, and processed for immunoprecipitation as described elsewhere. Protein concentration of the lysates was determined by the Bradford method before immunoprecipitation. Glucose consumption was assessed at the beginning and at the end of the stimulation and calculated as glucose consumption/mg of protein.

### Statistics

Data were tabulated and analyzed using SigmaPlot 12.5 statistical software. Data were analyzed for determining normal distribution with Shapiro-Wilk test. Mean comparison was performed with one-way Anova or unpaired Student's *t*-test. Statistical significance level was always set to *p* < 0.05.

### Study approval

For animal studies, all procedures were approved by the IACUC of the University of Catania. The use of patients' clinical data and CSF samples was approved by the Ethics Committee of Azienda Ospedaliera Universitaria (AOU) Careggi of Florence.

## Results

### Aß_1−42_ monomers interact with, and activate, type-I IGF receptors (IGF-IRs)

**Figure 1:**
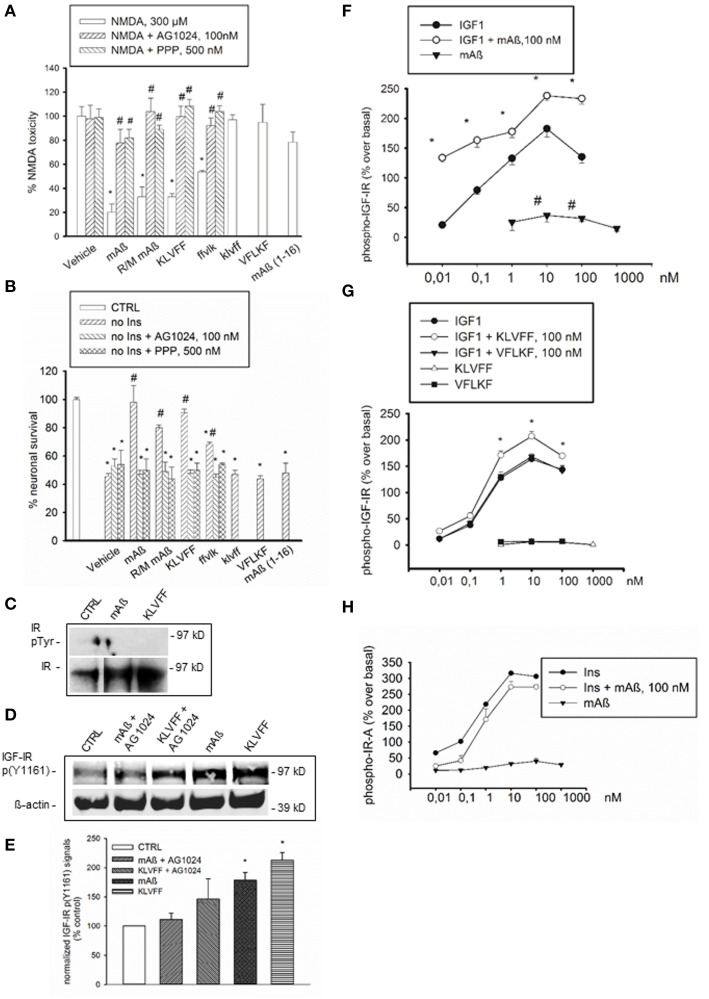
**Aß_1−42_ monomers activated IGF-IRs, but not IRs in primary neurons and recombinant cells**. The selective inhibitor of the IR superfamily, AG1024, and the preferential IGF-IR inhibitor, PPP, prevented the neuroprotective activity of Aß_1−42_ monomers, either human (mAß) or rat/mouse (R/M mAß), and of Ac-KLVFF-NH_2_ monomers (KLVFF) against NMDA-induced toxicity **(A)** or insulin-deprivation **(B)**. In **(A,B)** values are means ± S.E.M. of eight determinations from two independent experiments. Significantly different from NMDA (^*^) or from the respective peptide condition (#) at *p* < 0.05 (One-Way ANOVA + Fisher's LSD test). **(C)** Representative western blot analysis of immunoprecipitated IR beta subunit (IR) in neuronal extracts from control cultures (CTRL) or cultures exposed for 15 min to 100 nM of either monomeric Aß_1−42_ (mAß) or monomeric KLVFF peptide. IR bands are shown as control for loading. **(D)** Representative western blot analysis of IGF-IR p(Y1161) with the corresponding ß-actin. Data refer to neuronal extracts from control cultures (CTRL), or cultures exposed for 15 min to 100 nM of either monomeric Aß_1−42_ (mAß) or monomeric Ac-KLVFF-NH_2_ peptide (KLVFF) both in the presence and absence of AG1024 (100 nM). Identical results were obtained in experiments in which IGF-IR p(Y1161) was assessed in IGF-IR immunoprecipitates. **(E)** Densitometry of three separate western blots in which IGF-IR p(Y1161) signals were normalized either on ß-actin or total IGF-IR. Values (means ± S.E.M., *n* = 2−3) are expressed as % of the respective controls. ^*^p < 0.05 vs. CTRL by One-Way Anova + Fisher's LSD test. **(F)** Autophosphorylation of immunocaptured human IGF-IR in response to IGF1, mAß, or a combination of both. **(G)** Autophosphorylation of immunocaptured human IGF-IR in response to IGF1, KLVFF, or a combination of both. The lack of effects of the scrambled peptide, Ac-VFLKF-NH_2_ (VFLKF), is also shown. Both in **(F,G)** data are the means ± S.E.M. of three independent experiments and are expressed as % over basal receptor phosphorylation. ^*^Significant at *p* < 0.05 vs. IGF-1 alone, or vs. basal (#) (One-Way Anova + Fisher's LSD test). **(H)** Autophosphorylation of immunocaptured human IR-A in response to insulin (Ins), Aß_1−42_ monomers (mAß) or a combination of both. Data are representative of two experiments and are expressed as % over basal receptor phosphorylation. Phosphorylation was quantitated by ELISA as described under Methods.

We previously showed that monomeric human Aß_1−42_ protects cultured cortical neurons against excitotoxic death and death by trophic deprivation. Neuroprotection was abrogated by inhibitors of insulin/ IGF-1 receptor signaling (Giuffrida et al., 2009). Here, we found that neuroprotection was also produced by rat/mouse Aß_1−42_ (Figures 1A,B), which is resistant to oligomerization (Shivers et al., 1998). It was reasonable to hypothesize that the specific amino acid sequence engaged in the aggregation process could be required for neuroprotection. The 16–20 amino acid sequence of Aß_1−42_ (KLVFF) is critically involved in Aß_1−42_ oligomerization and is used as template for the design of beta-sheet breakers (Tjemberg, 1996). Synthetic Ac-KLVFF-NH_2_ maintained into a monomeric form (Supplemental Figure 1A) shared the protective activity of monomeric Aß_1−42_, and its action was prevented by the insulin/IGF-1 receptor inhibitor, AG1024, or by the selective type-1 IGF receptor (IGF-IR) inhibitor, PPP (Figures 1A,B). Neither the D-isomer, klvff (Supplemental Figure 1B), nor the scrambled peptide, VFLKF (Supplemental Figure 1D), induced neuroprotection. In contrast, the retroinverse ffvlk peptide (Supplemental Figure 1C), which maintains the overall spatial topology of KLVFF, was protective, albeit to a lesser extent. The human Aß_1−16_ and the Aß_17−42_ fragments were also tested. Monomers of Aß_1−16_, a fragment that is produced by combined α- and β- secretase activities (Nutu et al., 2013), were inactive (Figures 1A,B). The α secretase-generated Aß_17−42_, which is found in diffuse amyloid deposits and dystrophic neuritis associated with AD plaques (Higgins et al., 1996), could not be maintained in the monomeric conformation and rapidly precipitated into large aggregates (Supplemental Figure 1E). This was consistent with previous evidence showing that N-terminal deletions enhance peptide aggregation with respect to full-length Aß_1−42_ (Pike et al., 1995).

Based on neuroprotection data, we hypothesized that monomeric human Aß_1−42_ could interact with insulin/IGF-1 receptors *via* the 16–20 KLVFF sequence. Consistent with previous evidence (Zhao et al., 2008), a basal tyrosine-phosphorylation of the immunoprecipitated insulin receptor (IR) β subunit, which reflects the activated receptor, was virtually undetectable in cultured cortical neurons both in the absence and presence of either monomeric human Aß_1−42_ or Ac-KLVFF-NH_2_ monomers (Figure 1C). Because of the lack of detectable IR β phosphorylation, we could use an antibody directed against a phosphorylated tyrosine residue common to IGF-IR β and IR β [anti-p(Y1161) IGF-IR/p(Y1185) IR] to assess IGF-IR β phosphorylation without the need of immunoprecipitation. Both Aß and KLVFF monomers increased IGF-IR p(Y1161) levels. This increase was no longer visible in the presence of the receptor antagonist, AG1024 (Figures 1D,E). We searched for a direct peptide-receptor interaction using 3T3-like mouse fibroblasts with a disrupted IGF-IR gene and transfected with either human IGF-IR (R^+^ cells) or type-A IR (IR-A) (R^−^IR-A cells) cDNA (Pandini et al., 2002). On immunoadsorbed IGF-IRs derived from R^+^ cells, monomers of Aß_1−42_ or Ac-KLVFF-NH_2_ potentiated the ability of IGF-1 to promote autophosphorylation of the receptor-kinase domain (Figures 1F,G), with Aß_1−42_ also stimulating IGF-IR autophosphorylation in the absence of IGF-1 (Figure 1F). In contrast, Aß_1−42_ monomers had no effect on immunoadsorbed IR-A, both in the absence and presence of insulin (Figure 1H).

### Aß_1−42_ monomers stimulate glucose uptake in neurons by activating IGF-IRs

**Figure 2:**
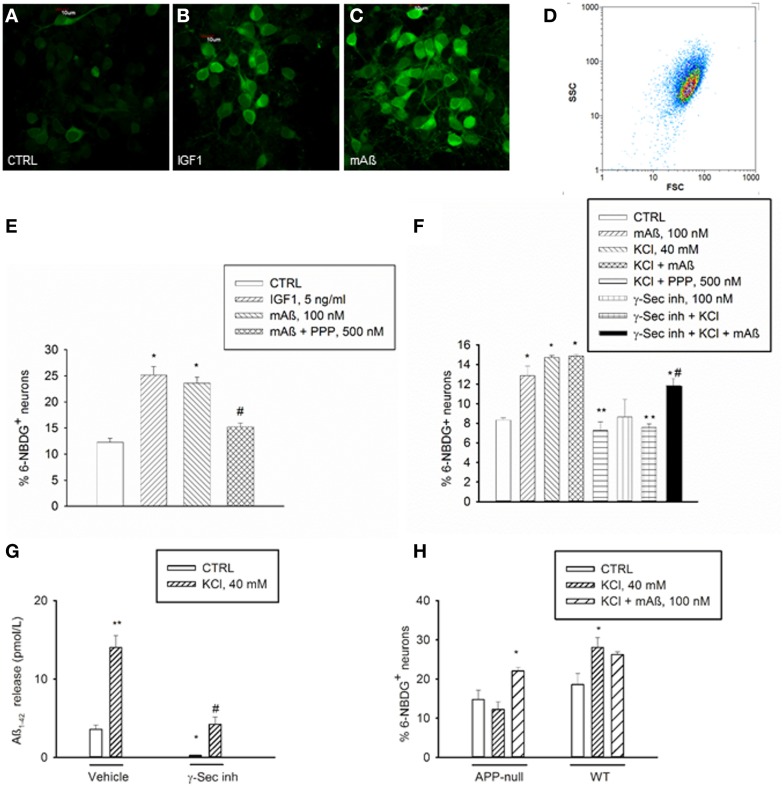
**Aß_1−42_ monomers stimulated glucose uptake in neurons by activating IGF-IRs**. Confocal images of 6-NBDG uptake in live neurons **(A)** exposed to either IGF-1 (5 ng/ml) **(B)** or monomeric Aß_1−42_ (mAß) **(C)** for 30 min after starvation, scale bar = 10 μm. **(D)** Representative scatter plot of the neuronal population examined by flow-cytometry. Percentage of 6-NBDG^+^ neurons following treatments was scored by flow cytometry in **(E,F)**. **(E)** The IGF-IR antagonist, PPP, prevented neuronal 6-NBDG uptake induced by Aß_1−42_ monomers (mAß). Values are means ± S.E.M. of two independent experiments. ^*^Significant at *p* < 0.05 vs. controls (CTRL), or vs. mAß(#) (One-Way Anova + Fisher's LSD test). **(F)** The IGF-IR antagonist, PPP, and the γ-secretase inhibitor IX (γ-Sec inh) prevented neuronal 6-NBDG uptake induced by a 15 min depolarization pulse with KCl. Values are means ± S.E.M. of two independent experiments. ^*^Significant at *p* < 0.05 vs. controls (CTRL), or vs. KCl (^**^), or vs. γ-Sec inh + KCl (#) (One-Way Anova + Fisher's LSD test). **(G)** γ-Secretase inhibitor IX blocked the endogenous production of Aß_1−42_ under both basal and depolarizing conditions. Values are means ± S.E.M. of three determinations from one representative experiment that was carried out under conditions similar to those utilized for the assessment of neuronal 6-NBDG uptake. γ-Secretase inhibitor IX (γ-Sec Inh) was present in the cultures for 2 h before washing and shifting into a glucose-free buffer for 30 min. Glucose-free buffer collected after 30 min, in the absence of γ-Sec inh., is reported as control condition. Where indicated, KCl was added 15 min before collecting the buffer. Aß_1−42_ was quantitated in the collected buffer by using the Wako human/rat Aß_42_ ELISA kit, high-sensitive. Significant at ^*^p < 0.05 and at ^**^p < 0.01 vs. control, and at #*p* < 0.01 vs. KCl alone (One-Way ANOVA + Fisher's LSD test). **(H)** Percentage of 6-NBDG^+^ neurons in APP-null cultures and related wt cultures. Values are means ± S.E.M. ^*^Significant at *p* < 0.05 vs. respective controls (CTRL) in the wt (*n* = 3−4) and in the APP-null condition (*n* = 6–9) (One-Way Anova + Holm-Sidak test).

IGF-1 is known to stimulate glucose uptake in neurons by mechanism(s) similar to those used by insulin in the periphery, including membrane translocation of glucose transporters (Gluts) (Bondy and Cheng, 2004). We deprived cultured neurons from glucose for 75 min prior to incubations with the fluorescent non-hydrolyzable glucose analog, 6-(N-(7-nitrobenzen-2-oxa-1,3-diazol-4-yl)amino)-6-deoxyglucose (6-NBDG) (Figure 2A). During starvation, neurons were exposed for 30 min to recombinant rat IGF-1 at concentrations (5 ng/ml) that selectively activate IGF-IR and are in the physiological range of concentrations in the CSF (Bilic et al., 2006). This treatment caused a significant increase in 6-NBDG uptake, as assessed by confocal microscopy or flow-cytometry (Figures 2B,E). Flow-cytometry showed a single cell population (Figure 2D) from which 6-NBDG^+^ neurons were scored. Consistent with its ability to engage IGF-IRs, monomeric Aß_1−42_ (100 nM) increased the population of 6-NBDG^+^ neurons following starvation, and its action was prevented by the IGF-IR inhibitor, PPP (Figures 2C,E). Thus, Aß_1−42_ monomers displayed IGF-1-like metabolic activity in cultured neurons. This, combined with the evidence that Aß_1−42_ is released from neurons in response to synaptic activity (Cirrito et al., 2005), provided the hint for testing the hypothesis that Aß_1−42_ monomers might function to increase glucose uptake during neuronal activation. A depolarization pulse with KCl (40 mM for 15 min) caused a significant increase in glucose uptake, which occluded any additional effect of Aß_1−42_ monomers (Figure 2F). Depolarization-induced glucose uptake was prevented by pretreatment with PPP (500 nM for 20 min), suggesting that endogenous activation of IGF-IRs was required for activity-dependent energy supply (Figure 2F). Addition of a γ-secretase inhibitor (γ-secretase inhibitor IX, 100 nM), which blocked the endogenous production of Aß_1−42_ (Figure 2G), blunted depolarization-induced glucose uptake, which was re-established by exogenous Aß_1−42_ monomers (Figure 2F). In neuronal cultures prepared from APP-null mice, KCl-induced depolarization failed to enhance glucose uptake unless exogenous Aß_1−42_ was added (Figure 2H).

**Figure 3:**
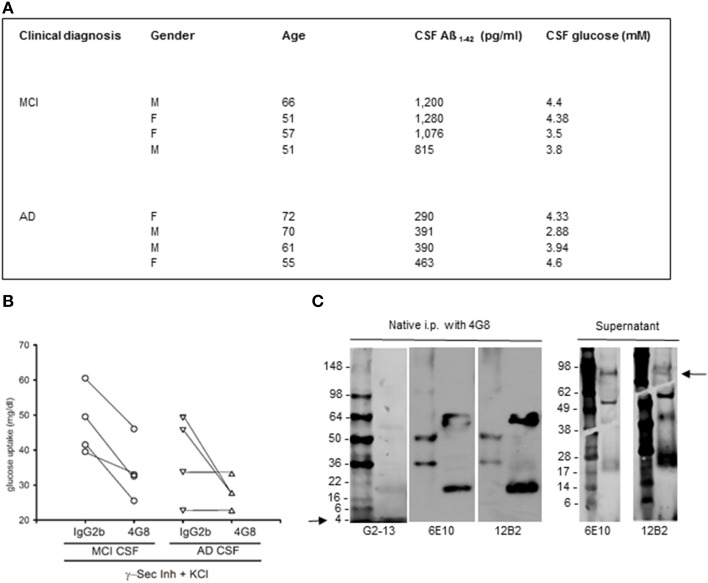
**Native human Aß stimulated glucose uptake in neurons. (A)** Summary of the cases used in this study. CSF samples were from the Department of Neurological and Psychiatric Sciences, University of Florence. Clinical diagnosis of AD was defined according to the *Diagnostic and Statistical Manual of Mental Disorders* criteria (DSM-IV). Clinical diagnosis of MCI was defined according to Petersen's validated criteria (Petersen et al., 2001). CSF Aß_1−42_ was quantitated by INNOTEST ß-amyloid (1–42) from Innogenetics. **(B)** Contribution of CSF Aß monomers to depolarization-induced neuronal glucose uptake both in the absence (IgG2b isotype control) and in the presence (4G8) of a neutralizing anti-Aß antibody. Neurons were pre-exposed to the γ-secretase inhibitor IX and underwent a 15 min depolarization pulse with 400 mM KCl (γ-Sec Inh + KCl). Mean values of glucose uptake (mg/dl) after the pulse were the following: 47.75 ± 4.77 vs. 33.87 ± 4.54^*^ (^*^different at *p* < 0.05, *n* = 4) in the MCI condition in the presence of IgG2b or 4G8, respectively; 38.13 ± 6.03 vs. 27.72 ± 2.14 (*n* = 4) in the AD condition in the presence of IgG2b or 4G8, respectively. Each n was run in duplicate and expressed as mean. **(C)** The ß-amyloid (17–24) 4G8 monoclonal antibody does not bind APP in human CSF samples under non-denaturing conditions. 200 ml of a MCI CSF sample, containing 1076 pg/ ml of Aß_1−42_, were treated with 4G8 antibody (Covance, 100 ng/50 ml) under native conditions for 2 h, before proceeding with the immunoprecipitation protocol. Western blot analysis was then performed both in the immunoprecipitated pellet (left) and in the supernatant (right). Three different antibodies were used to reveal APP and/or Aß in the samples. Both the 6E10 monoclonal antibody (Covance, 1:1000 dilution) and the 12B2 monoclonal antibody (IBL International, 5 mg/ml) failed to reveal the expected 100 kD band corresponding to APP in the pellet (left), but showed the APP band in the supernatant (right) (see arrow). Bands smaller than 100 kD, likely corresponding to Aß aggregates, were visible both in the supernatant and in the immunoprecipitated. An additional antibody, the G2-13 monoclonal antibody that specifically recognizes Aß_1−42_ at the C-terminus (Millipore, 1 mg/ml), was sensitive enough to detect the 4 kD Aß_1−42_ monomer at the electrophoresis front of the immunoprecipitated (left) (see arrow).

To examine whether native human Aß could share this activity, we carried out a set of experiments using CSF samples obtained from 4 MCI and 4 AD patients (Figure 3A). CSF levels of Aß_1−42_ in MCI patients (1032 ± 102 pg/ml) approximated the reference values of healthy individuals, whereas average levels in AD patients were about 3 fold lower (384 ± 36 pg/ml). CSF glucose contents varied among samples (Figure 3A), and were made uniform by addition of exogenous glucose. Cultured neurons, incubated with inhibitor IX to block the endogenous production of Aß_1−42_, were starved for 45 min and then incubated with human CSF. Glucose uptake stimulated by high K^+^ did not significantly differ between cultures incubated with MCI and AD CSF, although a trend to a reduction in AD-CSF treated cultures was seen (Figure 3B). We used the neutralizing 4G8 monoclonal antibody, which bound Aß but not APP in human CSF samples under non-denaturating conditions. Native CSF samples, as immunoprecipitated by 4G8 antibody and revealed by the G2-13 antibody, showed the 4 kD Aß_1−42_ monomer, but not the expected 100 kD band corresponding to APP (Figure 3C). Addition of the 4G8 antibody (100 ng/50 μl CSF) significantly reduced glucose uptake at least in cultures treated with MCI CSF (Figure 3B and related legend). This indicated that native human Aß was able to support glucose uptake in neurons.

### Aß_1−42_ monomers promote membrane translocation of Glut3 in neurons by activating IGF-IRs

**Figure 4:**
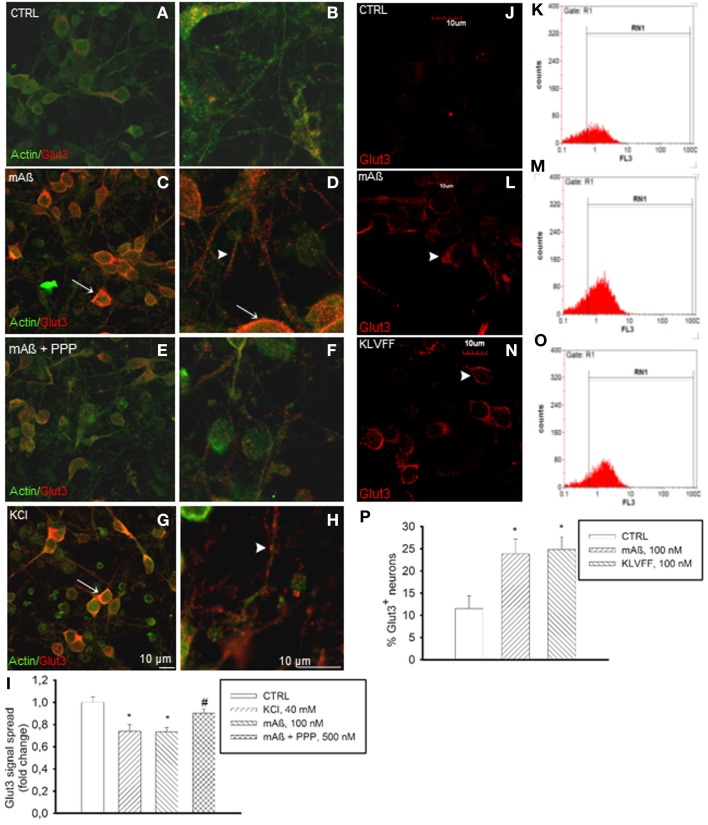
**Monomers of Aß_1−42_ and Ac-KLVFF-NH_2_ induced Glut3 translocation at neuronal plasma membranes**. Confocal images of neurons co-immunolabeled for Glut3 and ß-actin **(A,B)** following 10 min stimulation with 100 nM Aß_1−42_ monomers (mAß) both in the absence **(C,D)** and presence of the IGF-IR antagonist, PPP **(E,F)**, or with 40 mM KCl **(G,H)**. Before stimulations, neurons were glucose-deprived for 30 min. Arrows point to Glut3 immunoreactivity profiling neuronal perikarya in **(C,D,G)**; arrowheads point to Glut3 immunoreactivity profiling neuronal threads in **(D)** and **(H)**. Scale bars indicate low magnification **(A,C,E,G)** and high magnification **(B,D,F,H)** images. **(I)** Glut3 signal spread assayed in a z-stack series of neuronal slices as described under methods. Ten random fields for each experimental condition were imaged, and 10 neurons/field were scored. Each treatment was repeated twice in three separate experiments. Bars represent fold decrease of signal spread compared to controls (CTRL). ^*^Significant at *p* < 0.05 vs. controls (CTRL), or vs. mAß (#) (One-Way Anova + Fisher's LSD test). **(J,L,N)** Confocal imaging of the N-terminal extracellular domain of Glut3 in adherent non-permeabilized neurons. Immunostaining was barely visible under control conditions (CTRL). Arrowheads point to plasma membrane profiles in neurons that were exposed to 100 nM of either monomeric Aß_1−42_ (mAß) **(L)** or Ac-KLVFF-NH_2_ monomers (KLVFF) **(N)** for 10 min after glucose starvation. Scale bar = 10 μm. **(K,M,O,P)** Glut3 immunostaining carried-out in harvested non-permeabilized neurons and quantified by flow cytometry. Representative histograms displaying Glut3 fluorescence intensity (FL3) on the x-axis and the numer of events (counts) on the y-axis are shown for controls **(K)**, mAß **(M)**, and KLVFF **(O)**. The percentage of positive neurons is gated under RN1. **(P)** Bars represent means ± S.E.M of three determinations. 20,000 Neurons/determination were acquired. ^*^Significant at *p* < 0.05 vs. controls (CTRL) (One-Way Anova + Fisher's LSD test).

The membrane transporters, Glut3 and Glut4, mediate glucose transport in neurons (McEwen and Reagan, 2004). Glut3 has a neuropil localization (McEwen and Reagan, 2004), which might enable glucose transport in neurons to uphold glucose uptake during synaptic activity. Depolarization is known to promote the fusion of Glut3 vesicles with the cell surface in neurons (Uemura and Greenlee, 2006). In our cultures, K^+^-induced depolarization increased basal Glut3 immunoreactivity (Figures 4A,B) in neuronal threads, as well as in perikarya and axon hillocks (Figures 4G,H). Aß_1−42_ monomers induced a similar pattern of Glut3 immunoreactivity (Figures 4C,D), which was prevented by pretreatment with PPP (Figures 4E,F). The intense Glut3 immunoreactivity profiling neuronal perikaryon was paralleled by a reduction of Glut3 signal spread assayed in a z-stack series of neuronal slices (Figure 4I), suggesting that Aß_1−42_ monomers were promoting Glut3 translocation. Glut3 translocation was also assessed by immunolabeling neurons with an antibody raised against the exofacial epitope of Glut3 in the absence of membrane permeabilization. Both monomeric Aß_1−42_ and Ac-KLVFF-NH_2_ increased immunoreactivity for exofacial Glut3 as assessed by immunocytochemistry and cytofluorimetric analysis (Figures 4J–P). Consistent with the membrane translocation of Glut3, Aß_1−42_-induced glucose uptake occurred at concentrations (1.5 mM) at which only Glut3, which has the lowest K_m_ among all Gluts, is likely to function (Simpson et al., 2008) [extracellular glucose (mg/dl): 32.3 ± 4.7 and 18.7 ± 2–control vs. 100 nM Aß_1−42_, respectively; *n* = 4–5, *p* = 0.0127]. This putative Glut3-dependent glucose uptake was sensitive to the PI-3K-inhibitor, LY294002, [extracellular glucose (mg/dl): control (30.810 ± 1.975), 100 nM Aß_1−42_ (17.90 ± 0.473)^*^, 100 nM Aß_1−42_ + 10 μM LY294002 (26.950 ± 3.086)^*^; *n* = 4, ^*^p = 0.004 compared to the respective controls by One-Way Anova + Fishers' LSD], consistent with our previous demonstration that Aß_1−42_ monomers activate the PI-3K/AKT pathway in neurons (Giuffrida et al., 2009).

### Difference in IGF-IR response to Aß_1−42_ monomers between AD transgenic mice and wild-type littermates

We examined responses to IGF-1 and Aß_1−42_ monomers in forebrain slices prepared from AD mutant mice and their wt littermates. We used both 12-month old CRND8 mice, which show severe Aß plaque burden (Hanna et al., 2012), and 8-week old CRND8 mice, which show sporadic Aß deposits and perform normally (Chishti et al., 2001). At 12 months of age, physiological concentrations of exogenous IGF-1 (1 nM) enhanced Tyr-phosphorylation of IGF-IRs in wt but not in CRND8 slices. In contrast, exogenous monomeric Aß_1−42_ (100 nM) enhanced IGF-IR phosphorylation in CRND8 but not in wt slices. Basal glucose consumption was lower in CRDN8 slices and unresponsive to IGF-1. In contrast, monomeric Aß_1−42_ substantially increased glucose consumption in CRND8 slices. None of the treatments influenced glucose consumption in wt slices (Table 1). At 8 weeks of age, the IGF-IR response to either IGF-1 or Aß_1−42_ monomers was similar in forebrain slices of CRND8 mice and wt mice, as it was basal glucose consumption (Table 1).

**Table 1:** **Difference in the forebrain response to either IGF1 or Aß_1−42_ monomers between CRND8 transgenic and non-transgenic littermate mice**.

	**TgCRND8 (1 year old)**		**Non-Tg (1 year old)**	
	**p(Y1161) IGF-IR levels (% of basal)**	**Glucose consumption (μmol)/hr**	**p(Y1161) IGF-IR levels (% of basal)**	**Glucose consumption (μmol)/hr**
Basal	100	0.2 ± 0.02	100	0.35 ± 0.01
IGF1, 1 nM	88.5 ± 5.35	0.2 ± 0.018	153.467 ± 14.91^*^	0.35 ± 0.02
mAß, 100 nM	147.767 ± 4.68^*^	0.34 ± 0.014^#^	74.543 ± 12.25	0.35 ± 0.015
	**TgCRND8 (8 week old)**		**Non-Tg (8 week old)**	
	**p(Y1161) IGF-IR levels (% of basal)**	**Glucose consumption (μmol)/hr**	**p(Y1161) IGF-IR levels (% of basal)**	**Glucose consumption (μmol)/hr**
Basal	100	3.77 ± 0.322	100	3.71 ± 0.5
IGF1, 1 nM	129.333 ± 10.477^**^	3.42 ± 0.310	147.667 ± 9.735^**^	3.69 ± 0.798
mAß, 100 nM	125.333 ± 4.910^**^	3.03 ± 0.460	176.333 ± 7.446^**^	3.32 ± 0.381

Data were collected following ex-vivo stimulation of the brains, as described in the methods. p(Y1161)IGF-IR values (mean ± S.E.M., n = 3) are expressed as percentage of basal levels following normalization of the western blot hybridization signals. Different from its own basal at

* p < 0.05 or at

** *p < 0.001*.

*Glucose consumption was measured in the incubation buffer of the samples (n = 3 for 1 year old mice and n = 4 for 8 week old mice) at the beginning and at the end of the ex-vivo stimulation*.

# *Different from basal at p < 0.05 (mean ± S.E.M., n = 3)*.

### Peripheral IGF-1-like actions of Aß_1−42_ monomers

**Figure 5:**
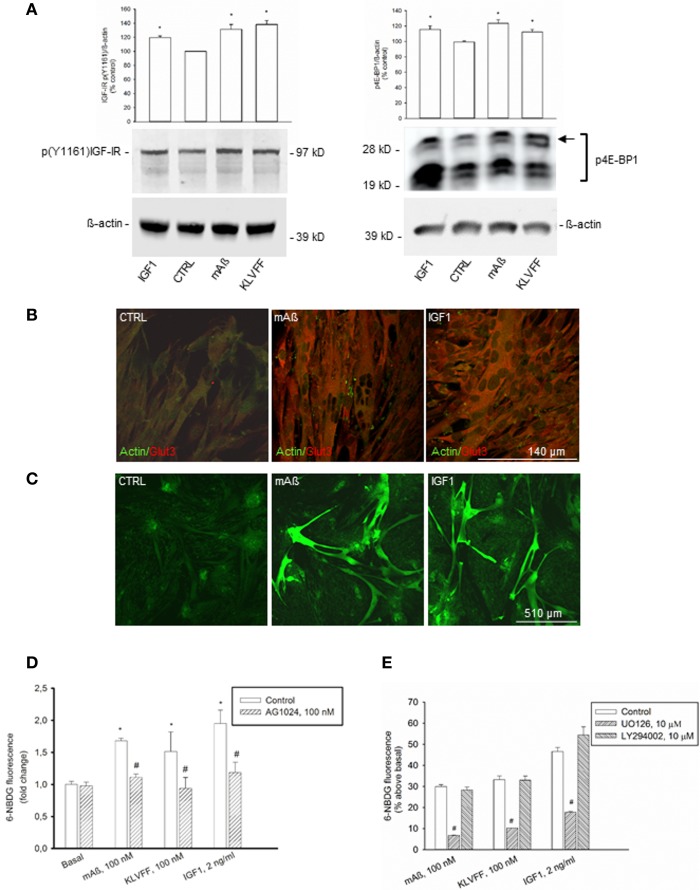
**IGF-1-like actions of Aß_1−42_ monomers in L6 rat skeletal myotubes. (A)** Left side: representative western blot of p(Y1161) IGF-IR in L6 extracts from control cultures (CTRL), or cultures exposed for 15 min to 100 nM of either monomeric Aß_1−42_ (mAß) or monomeric Ac-KLVFF-NH_2_ peptide (KLVFF). ß-actin bands are shown as control for loading. Bars refer to densitometric analysis of three separate western blots. Values (means ± S.E.M., *n* = 3) are expressed as % of the respective controls. ^*^p < 0.05 vs. CTRL by One-Way Anova + Fisher's LSD test. Right side: representative western blot analysis of phosphorylated 4E-BP1 (p4E-BP1) in L6 extracts from control cultures (CTRL) or cultures exposed for 15 min to 100 nM of either monomeric Aß_1−42_ (mAß) or monomeric Ac-KLVFF-NH_2_ peptide (KLVFF). Levels of high-molecular weight p4E-BP1 isoform (see arrow) were increased by either mAß or KLVFF. ß-actin bands are shown as control for loading. IGF-1 (2 g/ml) was used as a positive control within the experiments. Bars refer to densitometric analysis of three separate western blots. Values (means ± S.E.M., *n* = 3–4) are expressed as % of the respective controls. ^*^p < 0.05 vs. CTRL by One-Way Anova + Fisher's LSD test. **(B)** Aß_1−42_ monomers increased Glut3 immunoreactivity in L6 cells. Confocal images of L6 myotubes co-immunolabeled for Glut3 and ß-actin following 15 min stimulation with 100 nM Aß_1−42_ monomers (mAß) or 2 ng/ml IGF-1 are shown; scale bar = 140 μm. **(C–E)** Aß_1−42_ monomers promoted glucose uptake in L6 rat skeletal myotubes. Confocal images of 6-NBDG uptake in live L6 cells exposed to either monomeric Aß_1−42_ (mAß) or IGF-1 (2 ng/ml) for 15 min after starvation are shown in **(C)**; scale bar = 510 μm. In **(D)**, 6-NBDG fluorescence intensity was quantified by confocal imaging and represented as fold change with respect to basal. Fluorescence intensity was calculated from 300 cells/experiments in two independent experiments. ^*^Significant at *p* < 0.05 vs. basal, or vs. the respective control condition (#) (One-Way Anova + Fisher's LSD test). In **(E)** the mean 6-NBDG fluorescence intensity of the cell population was quantified by cytofluorimetric analysis. Bars represent means ± S.E.M of three-four determinations. 10,000 cells/determination were acquired. ^#^Significant at *p* < 0.05 vs. the respective control condition (One-Way Anova + Fisher's LSD test). UO126 and LY294002 were applied during starvation and throughout the course of the experiment.

To strengthen the evidence that monomeric Aß_1−42_ has IGF-1-like activity, we extended the analysis to classical peripheral actions of IGF-1. L6 rat skeletal muscle cells express both IGF-IRs (Beguinot et al., 1985) and Glut3 (Bilan et al., 1992). We assessed the phosphorylation state of the IGF-IR β subunit by using the antibody directed against the specific phosphorylated tyrosine residue, Y1161, common to the IR β subunit. Because the density IGF-I binding sites is one-hundred fold higher than the density of insulin binding sites in L6 cells (Beguinot et al., 1985), we used the anti-pY1161 antibody for the assessment of IGF-IR β phosphorylation without immunoprecipitation. In L6 myotubes, IGF-1 stimulated IGF-IR phosphorylation at concentrations as low as 2 ng/ml (Figure 5A). Both monomeric Aß_1−42_ and Ac-KLVFF-NH_2_ behaved similarly to IGF-I (Figure 5A), and enhanced the amount of phophorylated high-molecular weight eIF-4E binding protein, 4E-BP1 (Figure 5A), which is a downstream target of both the MEK/ERK and the PI-3K/AKT pathways (Kelleher and Bear, 2008). Similar to IGF-1, Aß_1−42_ monomers also increased Glut3 immunoreactivity in myotubes (Figure 5B). Monomeric Aß_1−42_ and Ac-KLVFF-NH_2_ (both at a 100 nM) stimulated 6-NBDG uptake as assessed by confocal microscopy (Figure 5C) and cytofluorimetric analysis (Figure 5D). Under all treatments, 6-NBDG uptake was prevented by the MEK inhibitor, UO126, but not by the PI-3K inhibitor, LY294002 (Figure 5E).

**Figure 6:**
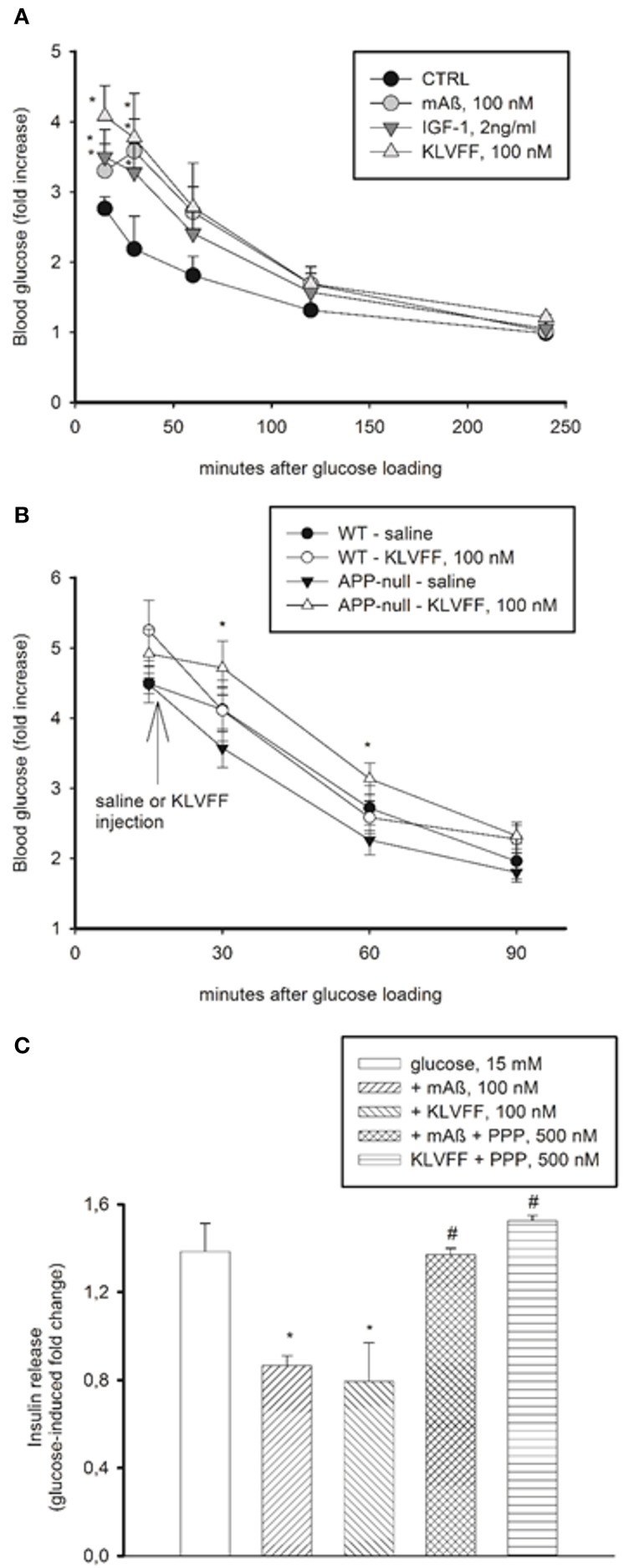
**Peripheral effects of Aß_1−42_ monomers**. **(A)** Aß_1−42_ monomers transiently increased blood glucose levels in CD1 male mice undergoing a glucose tolerance test (GTT). This effect was mimicked by the Ac-KLVFF-NH_2_ peptide and by IGF-1. Fasted mice were i.p. injected with either Aß_1−42_ monomers (mAß), KLVFF monomers or IGF-1 5 min before glucose loading (2 g/kg). Plots represent the fold increase of glucose levels over basal in 4 animals per experimental condition. ^*^Significantly different from control (CTRL) at *p* < 0.05 (One-Way Anova + Fisher's LSD test). Data are from one experiment repeated three times with similar results. **(B)** Ac-KLVFF-NH_2_ monomers sustained the rise in blood glucose levels in APP-null mice undergoing GTT. Mice, either C57BL/6J (WT) or APP-null, were i.p. injected with the Ac-KLVFF-NH_2_ peptide 15 min after glucose load. ^*^Significantly different from APP-null-saline at *p* < 0.001 (*n* = 4–10 animals/group, One-Way Anova + Fisher's LSD test). **(C)** Aß_1−42_ monomers reduced glucose-stimulated insulin release in pancreatic INS-1E cells. This effect was mimicked by the Ac-KLVFF-NH_2_ peptide and was prevented by the IGF-IR antagonist, PPP. Data, representative of three experiments, are fold change of glucose-stimulated insulin release. Significantly different from the 15 mM glucose condition (^*^), or the respective controls (#) at *p* < 0.05 and (One-Way Anova + Fisher's LSD test).

Physiological concentrations of IGF-1 inhibit insulin secretion from pancreatic ß cells (Van Schravendijk et al., 1990). Similarly to IGF-1, monomers of Aß_1−42_ and Ac-KLVFF-NH_2_ (injected i.p. to obtain plasma concentrations of 100 nM) caused a transient increase in blood glucose levels in CD1 mice undergoing a glucose tolerance test (GTT), which reflects the inhibition of insulin secretion (Figure 6A). Blood glucose levels during GTT were higher in the C57BL/6J strain than in the CD1 strain, consistent with the evidence that C57BL/6J mice have an inherited impaired glucose tolerance (Toye et al., 2005). In C57BL/6J mice, monomers of Ac-KLVFF-NH_2_ did not affect significantly blood glucose levels during GTT; however, in the congenic APP-null mice undergoing GTT, monomers of Ac-KLVFF-NH_2_ were able to sustain the rise in blood glucose levels when injected 15 min after glucose load (Figure 6B).

We also used INS-1E insulinoma cells, which secrete insulin in response to glucose (Hohmeier et al., 2000). We observed a 1.2–2 fold increase in insulin release by shifting the cells from low (3 mM) to high (15 mM) glucose concentrations. Monomers of Aß_1−42_ and Ac-KLVFF-NH_2_ inhibited glucose-stimulated insulin secretion, and the effect was prevented by PPP (Figure 6C). Thus, monomeric Aß_1−42_ and its functional epitope, KLVFF, exhibited IGF-1-like effects also outside the CNS.

## Discussion

The amyloid hypothesis postulates that Aß_1−42_, in a variety of aggregated forms, ultimately causes neurodegeneration in AD (Selkoe, 2011). However, drugs targeting Aß_1−42_ burden have been so far unsuccessful (Karran and Hardy, 2014), suggesting the need to explore the properties of the peptide beyond the process of aggregation. We have previously shown that monomeric Aß_1−42_ has a broad neuroprotective activity, which is sensitive to inhibitors of insulin/IGF-1 signaling (Giuffrida et al., 2009). Here we demonstrate that the KLVFF amino acid sequence of Aß_1−42_, which is recruited in the aggregation process, retained the neuroprotective properties of full-length Aß_1−42_. The additional findings that the retro-inverse pentapeptide (ffvlk) was neuroprotective, whereas the D-pentapeptide (klvff) was inactive, indicated a ligand-receptor interaction. IR and IGF-IR are homologous receptors, engaging many of the same intracellular pathways in the CNS and in the periphery (Belfiore et al., 2009). We have found that both monomeric human Aß_1−42_ and KLVFF selectively stimulated Tyr-phosphorylation of native IGF-IRs in primary cortical neurons. A direct activation of IGF-IRs by either monomeric Aß_1−42_ or KLVFF was demonstrated on immuno-captured IGF-IRs derived from recombinant cells. Under this condition, monomers of both Aß_1−42_ and KLVFF potentiated the ability of IGF-1 to promote autophosphorylation of the receptor-kinase domain, with Aß_1−42_ also displaying a low intrinsic efficacy. We concluded that monomeric Aß_1−42_ and KLVFF are able to interact with IGF-IRs, acting as positive allosteric modulators, and that the KLVFF sequence of Aß_1−42_ is involved in this interaction. Therefore, binding of Aß_1−42_ to IGF-IR may be lost when the KLVFF sequence becomes engaged in the formation of Aß_1−42_ aggregates.

There is evidence that the IGF-IR is resistant to IGF-1 activation in the AD brain (Talbot et al., 2012). Why the IGF-IR cannot be activated even by pharmacological doses of IGF-1 in AD brain is so far unexplained (Talbot et al., 2012). Our results obtained in brain tissue from CRND8 mice of different ages suggest that this could be due to the loss of Aß_1−42_ monomers accompanying the course of the pathology. In forebrain slices from old CRND8 mice with a severe Aß load, as opposed to slices obtained from young CRND8 brains, IGF-1 was unable to activate IGF-IRs, which, instead, remained sensitive to Aß_1−42_ monomers. We suggest that, by acting as an allosteric modulator, the exogenous monomer of Aß_1−42_ could be able to tune IGF-IRs in a way that cannot be accomplished by the orthosteric ligand, namely IGF-1 (De smet et al., 2014), and this effect would be unmasked under the condition of an advanced plaque burden and ensuing depletion of endogenous monomers. Whether or not the Aß_1−42_ monomer can activate native IGF-IR signaling in the absence of IGF-1, thus behaving as an allosteric agonist, remains to be established. In the brain, the endogenous tone of IGF-1 is like to decline with age (Piriz et al., 2011) and to rise in conjunction with pathological events (Cheng et al., 2000). The evidence that in old non-transgenic mice (i.e., in the aged-non injured brain), as opposed to young non-transgenic mice, Aß_1−42_ monomers failed to activate IGF-IRs suggests that, under our conditions, endogenous IGF-1 could be required for the activity of monomeric Aß_1−42_.

IGF-IRs and IRs control glucose uptake in neurons as they do in peripheral cells, i.e., by promoting membrane translocation of specific types of glucose transporters (Bondy and Cheng, 2004; McEwen and Reagan, 2004). Glucose uptake is particularly relevant during network activation, when activated neurons do not rely on astrocyte-released lactate to meet their energy demand (Ivanov et al., 2014). Neurons express Glut3, Glut4, and Glut8; among these, Glut4 is insulin-sensitive, but it is expressed only in few neuronal populations, whereas Glut8 never associates with the plasma membrane (McEwen and Reagan, 2004). We found that monomeric Aß_1−42_ and KLVFF were able to enhance glucose uptake in neurons *via* the activation of IGF-IRs, and this action was associated with membrane translocation of Glut3, which is localized mostly to the neuropil (McEwen and Reagan, 2004). In agreement with the evidence that Aß_1−42_ monomers activate the PI-3K/AKT pathway in neurons (Giuffrida et al., 2009), the PI-3K-inhibitor, LY294002, prevented glucose uptake induced by monomeric Aß_1−42_. More important, we demonstrated that Aß_1−42_ was necessary for activity-dependent glucose uptake in neurons. In fact, a depolarizing pulse of K^+^ ions was unable to stimulate glucose uptake if endogenous production of Aß_1−42_ was blunted by an inhibitor of γ-secretase or by the genetic deletion of APP. Under both circumstances, depolarization-induced glucose uptake was rescued by exogenous application of monomeric Aß_1−42_, either synthetic or derived from human CSF samples. Although synthetic Aß differs from natural Aß in terms of effective concentrations (Reed et al., 2011), our data demonstrated that the effects of the synthetic Aß monomer were reproduced by the endogenous rat peptide and by the human-derived peptide.

Monomeric Aß_1−42_ and KLVFF exhibited IGF-1-like activity also in peripheral cells, including pancreatic β cells and skeletal muscle cells. In both systems, as in neurons, the endogenous tone of IGF-1 (Yang et al., 2002; Oksbjerg et al., 2004; see also neuronal cultures in the methods section) was likely to be relevant for the action of KLVFF, but could be dispensable for the action of Aß_1−42_ monomers, which, at least in recombinant R^+^ cells, displayed a small agonist activity. In skeletal muscle cells, monomeric Aß_1−42_ and KLVFF reproduced the intrinsic signaling properties of IGF-1, including the phosphorylation of the protein synthesis regulator, 4E-BP1, membrane translocation of Glut3, and MEK/ERK-dependent glucose uptake. This last finding was consistent with the evidence that, in L6 skeletal muscle cells, Glut3 depends on the MEK/ERK pathway for membrane translocation (Taha et al., 1997). Overall, these data strengthen the evidence that Aß_1−42_ monomers were acting on IGF-IRs, suggesting a possible contribution of monomers to the control of peripheral glucose homeostasis.

Accordingly, due to the ability to inhibit insulin secretion, monomers of both Aß_1−42_ and KLVFF raised blood glucose levels in outbred mice injected i.p. with a glucose load. We did not observe this effect in inbred mice with an inherited impaired insulin release (Toye et al., 2005), unless they were carrying a genetic deletion of APP. Hence, the condition of constitutive glucose intolerance could determine a desensitized response to amyloid, which was maintained instead in APP-null mice lacking endogenous amyloid. Although these data are a corollary of the main finding that monomeric Aß_1−42_ acts as a positive allosteric modulator of IGF-IRs, they point to potentially relevant glucoregulatory properties of peripheral amyloid deserving further investigation.

With regard to the central IGF-1-like metabolic actions of Aß_1−42_ monomers, our data hint at the relevance of a loss of Aß_1−42_ monomers in the pathophysiology of AD. FDG-PET studies have shown low rates of brain glucose metabolism in young adult at genetic risk of late-onset AD (Reiman et al., 2004), and in presymptomatic individuals with familial AD (Mosconi et al., 2006). FDG autoradiography in transgenic AD mice has demonstrated a pattern of progressively reduced metabolic activity similar to that observed in human AD (Reiman et al., 2000). The profound resistance of the AD brain to IGF-1 (Talbot et al., 2012), which has been replicated in AD transgenic mice (Zhang et al., 2013 and present manuscript), could account for the observed glucose metabolic deficits (Reiman et al., 2000; Caselli et al., 2008). This suggestion is consistent with the evidence that glucose metabolism in most neurons is insulin insensitive (Lucignani et al., 1987; Talbot et al., 2012) and, instead, regional glucose utilization parallels IGF-1 and IGF-IR expression at least in the developing brain (Cheng et al., 2000). We propose that the impairment of brain glucose uptake, preceding the clinical onset of AD by years, could be due to the early process of amyloid aggregation causing pauperization of Aß monomers, which normally modulate IGF-IRs. Over the years, amyloid load could progressively overcome the metabolic reserve that supports cognitive function (Stranahan and Mattson, 2012) leading to the onset of dementia. Finally, the evidence that brain metabolism in AD patients is more severely impaired during activation than at rest (Kessler et al., 1991) is consistent with the hypothesis that the reduced neuronal secretion of Aß occurring with aging (Cirrito et al., 2003; Tampellini et al., 2011) might impair the brain ability to cope with transient needs in neuronal energy provision.

## Author contributions

MG, MT, G. Pandini, and FC were responsible for the design, acquisition, analysis, interpretation, and approval of the final version of the manuscript; GB, CB, PD, G. Pappalardo, FA, SC, SB, and BN were responsible for acquisition, analysis, interpretation, and approval of the final version of the manuscript; SS, RV, and ER were responsible for the design, analysis, interpretation, and approval of the final version of the manuscript; FN and AC were responsible for the design, analysis, interpretation, drafting, and approval of the final version of the manuscript.

### Conflict of interest statement

The authors declare that the research was conducted in the absence of any commercial or financial relationships that could be construed as a potential conflict of interest.
