# Supplementary material for: Monomeric ß-amyloid interacts with type-1 insulin-like growth factor receptors to provide energy supply to neurons
Source: Front Cell Neurosci. 2015 Aug 7;9:297. doi: 10.3389/fncel.2015.00297 (PMC4528168; doi:10.3389/fncel.2015.00297)
Supplement: Supplementary file 1 [file Image1.PDF]

Supplementary Material

MONOMERIC ß-AMYLOID INTERACTS WITH TYPE-1 INSULIN-LIKE GROWTH FACTOR RECEPTORS TO PROVIDE ENERGY SUPPLY TO NEURONS

Maria Laura Giuffrida[1,9], Marianna Flora Tomasello[1,2,9], Giuseppe Pandini[1,3], Filippo Caraci[4,5], Giuseppe Battaglia[6], Carla Busceti[6], Paola Di Pietro[6], Giuseppe Pappalardo[1], Francesco Attanasio[1], Santina Chiechio[4], Silvia Bagnoli[7], Benedetta Nacmias[7], Sandro Sorbi[7], RiccardoVigneri[1,3], Enrico Rizzarelli[1], Ferdinando Nicoletti[6,8], Agata Copani[1,4*]

[1]National Research Council, Institute of Biostructure and Bioimaging, 95125 Catania, Italy.

[2]PhD Program in Neuropharmacology, University of Catania, 95125 Catania, Italy.

[3]Department of Clinical and Molecular Biomedicine, University of Catania, 95125 Catania, Italy.

[4]Department of Drug Sciences, University of Catania, 95125 Catania, Italy.

[5]IRCCS Associazione Oasi Maria S.S., Institute for Research on Mental Retardation and Brain Aging, 94018 Troina, Enna.

[6]I.R.C.C.S Neuromed, 86077 Pozzilli, Italy.

[7]NEUROFARBA, University of Florence, 50139 Florence, Italy.

[8]Department of Human Physiology and Pharmacology, University "La Sapienza", 00185 Rome, Italy.

[9]Equally contributed to the work.

*** Correspondence:** acopani@katamail.com

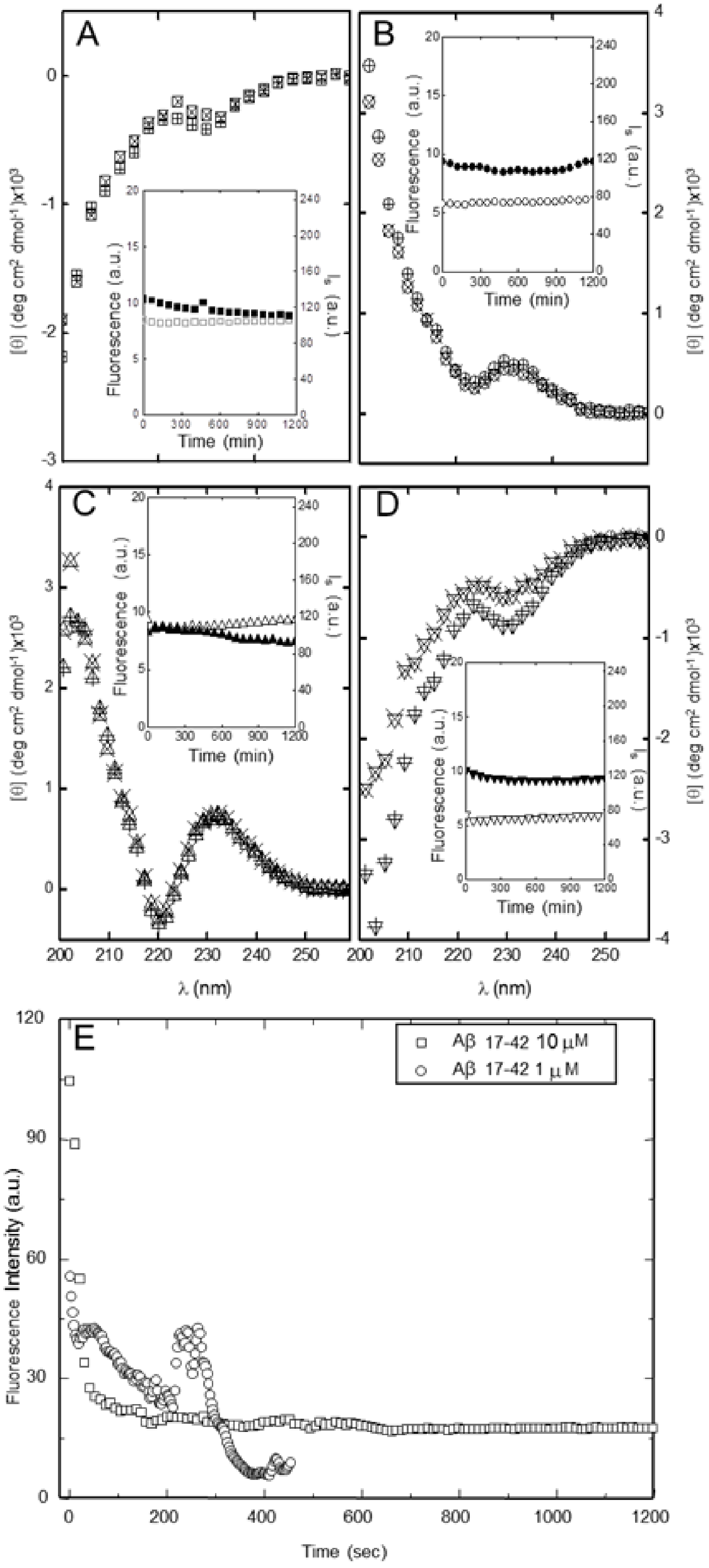
A
B
C
D
E
Fluorescence (a.u.)
Time (min)
I_s (a.u.)
[θ] (deg cm² dmol⁻¹)x10³
λ (nm)
Aβ 17-42 10 μM
Aβ 17-42 1 μM
Fluorescence Intensity (a.u.)
Time (sec)

Supplementary Figure 1. Pentapeptides (A-D) maintained a monomeric, yet flexible conformational state overtime. Circular Dichroism spectra of (A) KLVFF (square), (B) klvff (circle), (C) ffvlk (triangle up) and (D) VFLKF (triangle down) peptide solutions, incubated at 37 °C, at t = 0 min (x symbol) and t = 1200 min (+ symbol), respectively. All the CD spectra exhibited dichroic bands (either positive or negative depending on the peptide stereo-configuration) around 200 - 230 nm, suggesting that the pentapeptides did not adopt any preferred secondary structure in aqueous solution. Inset: ThT fluorescence intensities (open symbol) at 480 nm and Rayleigh scattering intensities (filled symbol) at 400 nm as a function of time for peptide samples incubated at 37 °C. Both Th-T fluorescence and Rayleigh scattering measurements confirmed the un-aggregated condition and featureless conformational state of the peptide chains. $Aß_{17-42}$ samples (E) failed to maintain the monomeric state. Freshly prepared samples were dissolved in 10 mM phosphate buffer pH=7.2 containing Th-T in a fivefold excess with respect to $Aß_{17-42}$ concentration. The high Th-T fluorescence intensity values that were observed soon after peptide dissolution indicated a rapid formation of amyloid aggregates at both 1 and 10 μM concentrations; the rapid Th-T fluorescence decay indicated, instead, peptide precipitation into large aggregates.
